# Outlines of a Course of Lectures on the Principles and Practice of Surgery

**Published:** 1858-09

**Authors:** 


ART. V.—*Outlines of a Course of Lectures on the Principles and Practice of Surgery,* delivered by E. GEDDINGS, M.D., Professor of Surgery in the Medical College of the State of South Carolina. Prepared by THOS. S. WARING, M.D., and SAMUEL LOGAN, M.D., from notes taken during the course. Published with the consent of and revised by Professor GEDDINGS. 8vo., pp. 560. Charleston: S. G. Courtenay & Co. 1858.

THE May number of the *Charleston Medical Journal and Review* announced the resignation of Professor Geddings, of the chair he had so long held in the Medical College of the State of South Carolina. After having had, what the same journal, in a biographical sketch of the Professor contained in the number for May, 1857, styles the "proud satisfaction" of receiving the first degree at the first commencement of that institution, in the spring of 1825, he was connected with it for some time as demonstrator of anatomy, and in 1837 he was appointed to fill one of its chairs. Professor Geddings had then been associated, for many years, with the medical school in Charleston; and we have the authority of the journal cited above, for declaring that it has owed to his labors and name a large measure of its popularity and influence.

The volume before us was published with the hope, as expressed by Professor Geddings himself, in a few prefatory remarks, that it might prove useful to his pupils as furnishing an outline of his course of lectures, which could be filled up by them while listening to his instructions; and also with the hope that, among the numerous alumni of the Charleston school, it might recall associations upon which he, at least, should always look back with pleasure. The first of these hopes has, of course, been frustrated by his resignation; the second will doubtless be fulfilled; in fact, the step he has thought fit to take must naturally enhance the value of this publication to his former pupils.

This work is one which will be esteemed more particularly by those who have listened to Professor Geddings's instructions; but, from the very respectable reputation of that surgeon, it also possesses interest for the profession at large. No one can expect to find therein, in an abstract of sixty-eight lectures, an approach to a complete treatise on the principles and practice of surgery. Delivered without notes, and abundantly illustrated, this abstract must fall much short of the lectures themselves; yet it can be taken, according to the Professor's own statement, as a fair and faithful exposition of the facts and principles of surgery, which he has taught for many years. Some of the omissions have been supplied by foot notes, and by ten extended essays, contributed by the editors.

This course of lectures is divided into three parts, which are thus defined in the editorial preface:—

*Part I.* Is devoted to the discussion of those affections of a strictly *constitutional* character, which, though often resulting in local complications, are not necessarily confined to any particular tissue or locality; such as inflammation, syphilis, and the like.

*Part II.* Refers to all those affections, accidents, or operations which appertain to active *structures* or *tissues* only; these affections, operations, etc., being *particular in kind*, but occurring in many different portions of the human frame; such as fractures, aneurism, etc.

*Part III.* Comprehends all accidents, affections, and operations of a *strictly local* character; this division being, as the Professor calls it, *Regional or Topographical Surgery.* Compression and concussion of the brain, ozœna, hare-lip, tracheotomy, etc. etc., come under this head.

It seems to us that a course of lectures on surgery should always contain a fourth part, devoted to general considerations on surgery, by which the course should be opened. We mean that there should be a part wherein surgery is defined, and its end and object stated, when general directions as to surgical diagnosis and therapeutics are given, with the rules to be observed in all operations. In this part, the minor operations, cauterizations, local and general bleedings, the application of the seton and the moxa—in a word, minor surgery should be treated of. There may have been some good reason for making this omission, but no reference is made to it, and we think it one to be remarked.

If an attempt had not been made by Drs. Waring and Logan to supply omissions, we should pass over all others; but, under the circumstances, it would be wrong not to point out some of those found in the work, that our readers may judge how very imperfect it is. In so doing, we do not wish to be misunderstood; we are aware that the publication makes no claim to be a complete treatise on the principles and practice of surgery, and we are desirous of making every allowance for its deficiencies.

There is nothing in the work upon varix, or any of the affections of veins, except a few remarks upon varicocele, and upon arterio-venous aneurism of the elbow. Not a word is said about tubercular affections which require surgical treatment, unless it be a short chapter on what is badly styled lymphatic swelling and abscess. In a long essay on diseases about the anus, there is not a word in regard to fissure of the anus. In the article upon vesico-vaginal fistulæ, the cauterization of Dupuytren, and the barbarous operations of Jobert are commended, while neither Sims nor Bozeman are at all mentioned. Tetanus is not treated of, though so commonly met with in Charleston, and in that section of country where this work will principally be read. Diseases and injuries of the eye-ball and its appendages are not mentioned, with the exception of a few words upon syphilitic iritis, in the essay upon syphilis; nor is anything said of affections of the vertebral column.

The sins of commission of the editors, or what we esteem to be such, we shall be still more brief in noting. We must express our astonishment to see it taught at the present day, and by a young man, that gonorrhœa, "though altogether local at first, very soon becomes constitutional," (p. 524;) and it is with sincere regret that we find it advised in the treatment of primary or local syphilis, "to adopt a constitutional treatment in conjunction with the local from the first," (p. 70.) A chancre may or may not be followed by constitutional syphilis. By giving mercury, you may, therefore, subject your patient unnecessarily to its debilitating influence, and, what is very important, you place him in such a condition that he does not really know whether he has syphilis in his system or not; for mercury does no more than retard the breaking out of the symptoms. We should always wait for a constitutional symptom to declare itself, and we lose nothing by so doing.

Though the work has been revised by Professor Geddings, we feel more inclined to charge typographical errors upon the editors. Mistakes in spelling proper names are numerous. We find *Physic* at page 411, and, at page 474, *Physick;* at pages 199 and 220, and in the index, we find *Dessault;* at page 139 *Ambrose Pare*, and at page 305 *Parê; Sedillot, Berard*, and *Negrier*, at pages 47, 49, and 393, should have, of course, the accent over the first *e;* we find *subsaltus tendinum* at page 47, and in the prescription at page 71, *vini. rubri.* These notices are sufficient to show that proper care has not been taken in the publication of these lectures.

As far as can be judged of them from this work, the surgical principles, and the practice also, which were taught by Professor Geddings, are very correct. What is most particularly to be commended, is the care manifested to teach his pupils not to endanger the lives of their patients by attempting too much. In some particular points, his practice is not that which we like best ourselves. For instance, in amputations the flap operation is preferred by him to the circular. So far as the safety of the patient is concerned, he declares that there is no difference whatever; it is only a matter of taste with the surgeon, (p. 251.) We are satisfied ourselves, that in the continuity of the limbs, the circular operation should always be performed, when the injury will admit of it. By this method the wound inflicted by the knife is always less extensive; and in dressing it, analogous parts can be placed in contact with each other, and the cicatrice is in the centre of the stump, which is by far the best place for it. In regard to the length of time after dislocation at which it is proper to attempt reduction, three months for the humerus, and six weeks for the femur, are set down as the utmost limits within which an attempt at reduction should be made, (p. 197.) Now it is not right to fix a time in this manner, so much for each joint, without paying attention to the peculiarities of the particular case. The time must vary in every case of dislocation, and the causes of the variation are the inflammation following the injury, and the degree of displacement. A dislocation, followed by violent inflammation will, when old, present more resistance than one which had not been followed by inflammation. The degree of displacement is a still more essential point. Malgaigne declares that he would not hesitate to attempt the reduction of an incomplete luxation of the humerus at the end of a year, of a sub-coracoid luxation at the end of six months and more; while, after four or five months, an intra-coracoid luxation would leave him but little hope, and he would have still less, after two or three months, for a sub-clavicular luxation. (*Traité des Frac. et des Lux.* Tome ii., p. 187; Paris, 1855.)

In the operation of lithotrity, the Professor teaches that, as regards the condition of the bladder, it is a matter of no consequence whether it be full or empty, (p. 547.) Certainly there is much less risk of seizing the mucous membrane of the bladder when it is distended; and we have always seen Civiale, and other most experienced operators, fill the bladder with warm water before operating. In the treatment of aneurism, the ligation of the artery is taught to be far preferable to the application of compression. This question has, however, been treated of at length in this journal, in the May number for 1857, and we will not enter into it here.

